# Supplementary material for: MarsGT: Multi-omics analysis for rare population inference using single-cell graph transformer
Source: Nat Commun. 2024 Jan 6;15:338. doi: 10.1038/s41467-023-44570-8 (PMC10771517; doi:10.1038/s41467-023-44570-8)
Supplement: Supplementary file 1 — Supplementary Information [file 41467_2023_44570_MOESM1_ESM.pdf]

Supplementary files of

MarsGT: Multi-omics analysis for rare population inference using single-cell graph transformer

Xiaoying Wang[1,2,3,*], Maoteng Duan[1,*], Jingxian Li[1], Anjun Ma[2,3], Gang Xin[3], Dong Xu[4,5], Zihai Li[3], Bingqiang Liu[1,$], Qin Ma[2,3,$]

[1] School of Mathematics, Shandong University, Jinan, Shandong, 250100, China

[2] Department of Biomedical Informatics, College of Medicine, The Ohio State University, Columbus, OH, 43210, USA

[3] Pelotonia Institute for Immuno-Oncology, The James Comprehensive Cancer Center, The Ohio State University, Columbus, OH 43210, USA

[4] Department of Electrical Engineering and Computer Science, University of Missouri, Columbia, MO 65211, USA

[5] Christopher S. Bond Life Sciences Center, University of Missouri, Columbia, MO 65211, USA

[*] These authors contributed equally

[$] These authors jointly supervised this work:

Bingqiang Liu: bingqiang@sdu.edu.cn

Qin Ma: qin.ma@osumc.edu

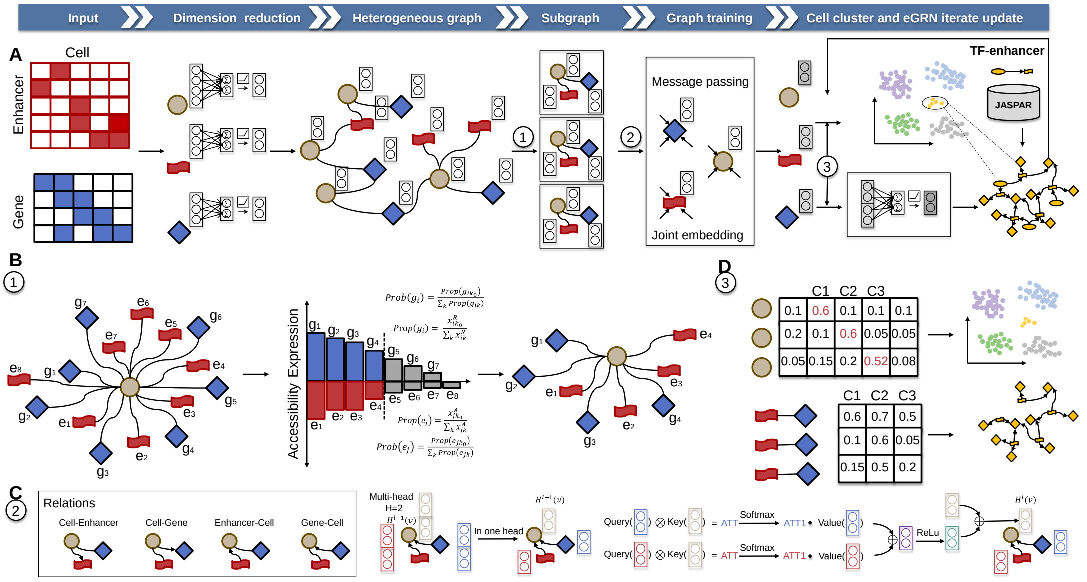

Supplementary Fig. 1. The detailed workflow of MarsGT for rare cell population identification. (A) The overall framework of MarsGT. Five main steps were included in carrying out cell clustering and gene regulatory networks from the matched scRNA-seq and scATAC-seq data. First, the scRNA-seq and scATAC-seq are used for dimension reduction by linear layer and ReLU layer, and then the initial embedding of cells (circle), peaks (flag), and genes (rhombus) are obtained. Second, a heterogeneous graph is comprised of cells, genes, peaks, and initial embeddings. Then, the probability-based subgraph sampling is designed to extract rare signals and for large graph training. For the graph training process, the embeddings of all nodes are updated based on the attention-based message passing operation. Based on the updated embedding, the cell clusters and gene regulatory relations are predicted. (B) Subgraph sampling step, genes/peaks will be selected for the cell if the genes/peaks are highly

accessible/expressed in the cell and lowly accessible/expressed in the other cell. (C) In the graph training step, four relations of the graph are used to pass the message. Arrows represent the connection between a target node and source nodes. The right panel shows the attention mechanism. (D) The final output of the MarsGT is the cell clusters, calculated by the cell probability matrix, and regulatory relations, calculated by the peak-gene link probability matrix.

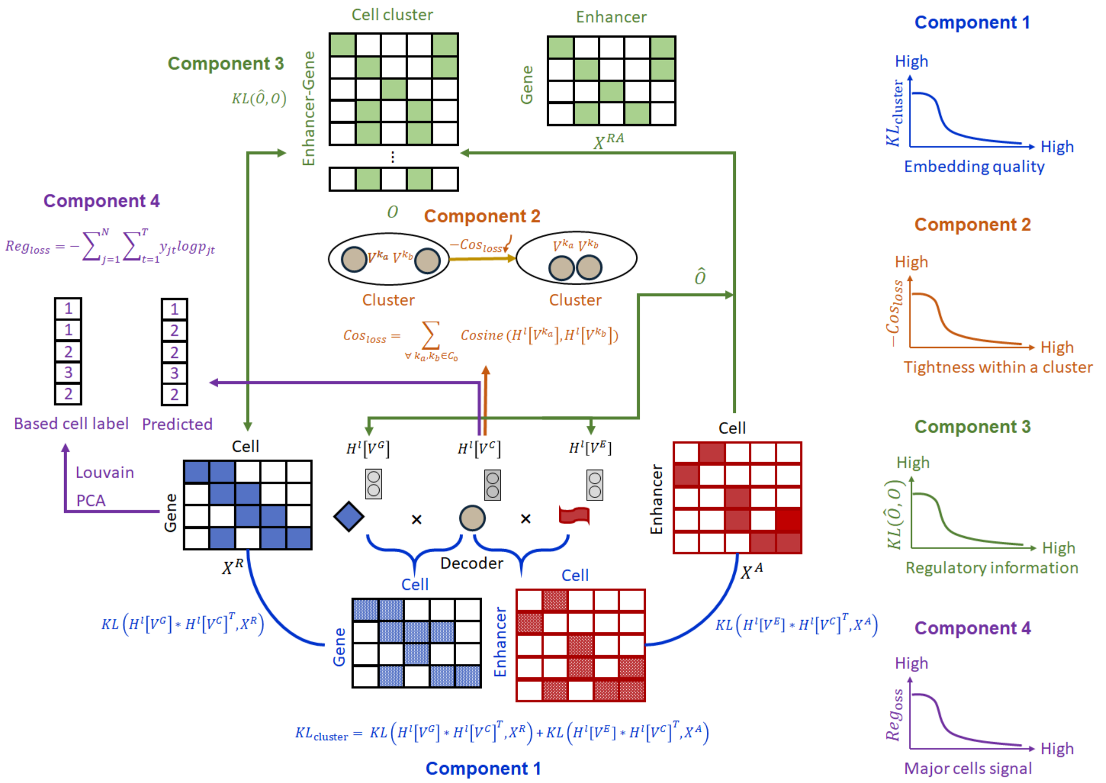

Supplementary Fig. 2. The detailed loss function explanation of MarsGT for rare cell population identification. Component 1: gene embeddings and peak embeddings interact through an inner product operation with cell embeddings, decoding the encoded information and preserving the original data (Blue color). Component 2: the cosine similarity of cell embeddings is calculated to ensure that embeddings within the same cluster are closer or more tightly knit. (Orange color). Component 3: The cell gene embeddings and peak embeddings are integrated and utilized as the input for the peak-gene relation predictor, which outputs a peak-gene link probability matrix. Subsequently, the KL divergence is calculated to ensure that the union of peak-gene relations under the predicted cell cluster is similar to the peak-gene relations of bulk level, and to ensure that if a peak regulates a gene under a cell type, then the peak is accessible and the gene is expressed (Green color). Component 4: scRNA-seq data is employed to compute the base cell cluster labels, preserving the primary cell signals (Purple color).

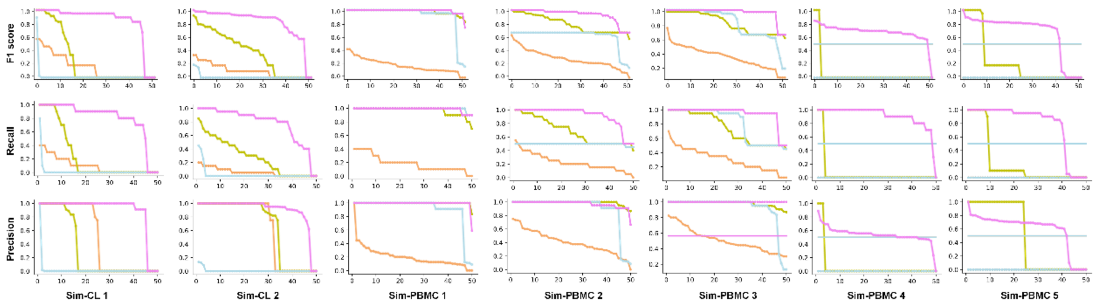

Supplementary Fig. 3. Performance comparison of rare cell identification on 350 original simulated datasets in terms of F1 score, Precision, and Recall.

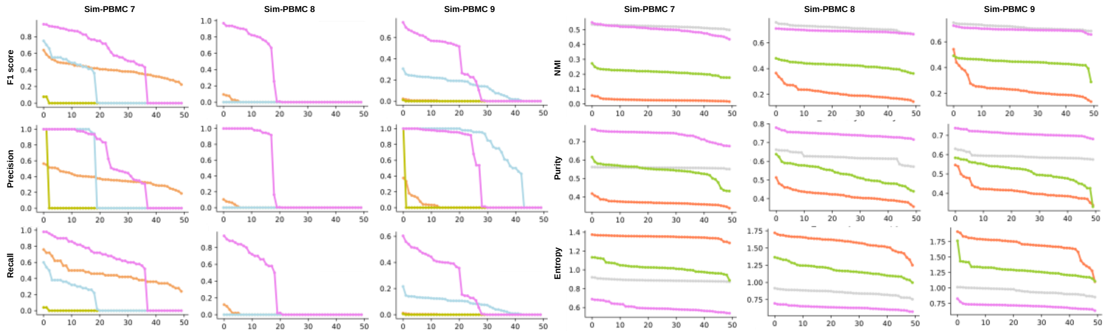

Supplementary Fig. 4. Performance comparison of the major cell and rare cell population simultaneously identification ability on 150 new simulated datasets in terms of F1 score, Precision, Recall, NMI, Purity, and Entropy.

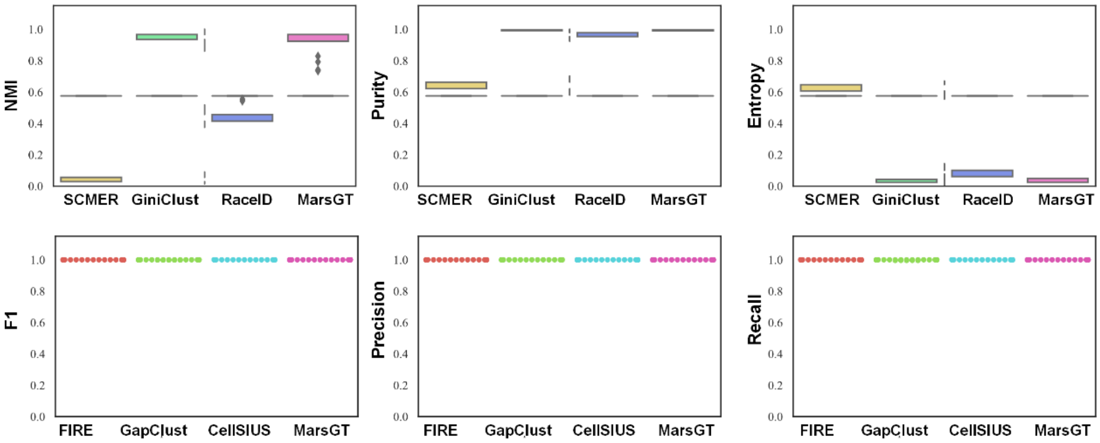

Supplementary Fig. 5. Performance comparison of the false positive rate on 50 simulated datasets in terms of F1 score, Precision, Recall, NMI, Purity, and Entropy. Each box showcases the minimum, first quartile, median, third quartile, and maximum evaluation scores in 50 simulation datasets (n = 50).

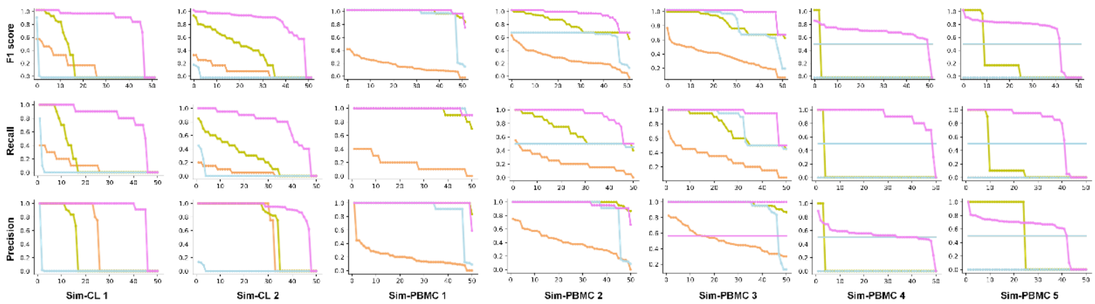

Supplementary Fig. 6. Performance comparison of the major cell and rare cell population simultaneously identification ability on 350 original simulated datasets in terms of NMI, Purity, and Entropy.

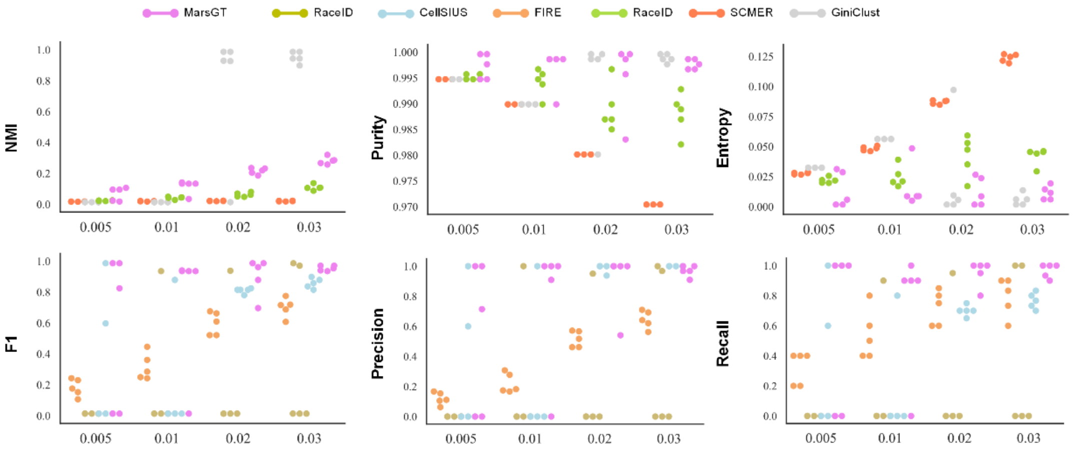

Supplementary Fig. 7. Performance comparison of the different proportion rare cell population identification ability on 5 simulated datasets in terms of F1 score, Precision, Recall, NMI, Purity, and Entropy.

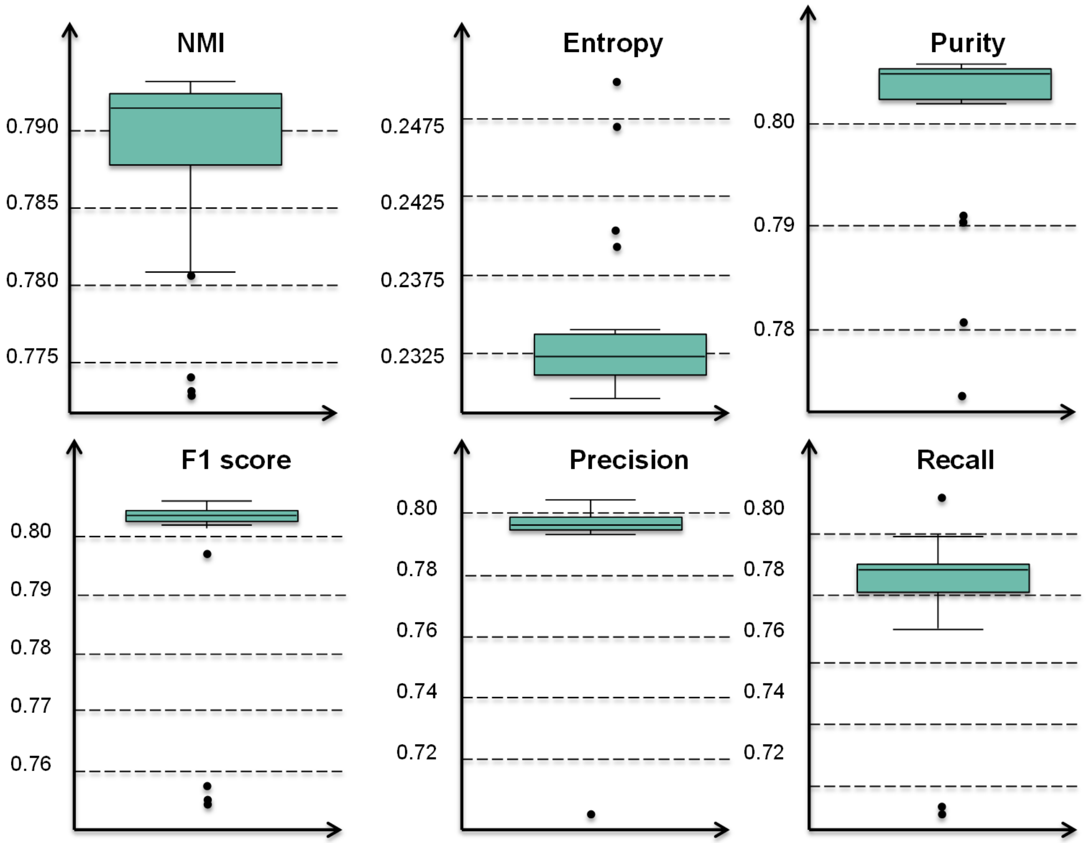

Supplementary Fig. 8. Robustness test. MarsGT runs 20 times on the independent test set. Each box showcases the minimum, first quartile, median, third quartile, and maximum evaluation scores in 20 time runs (n = 20).

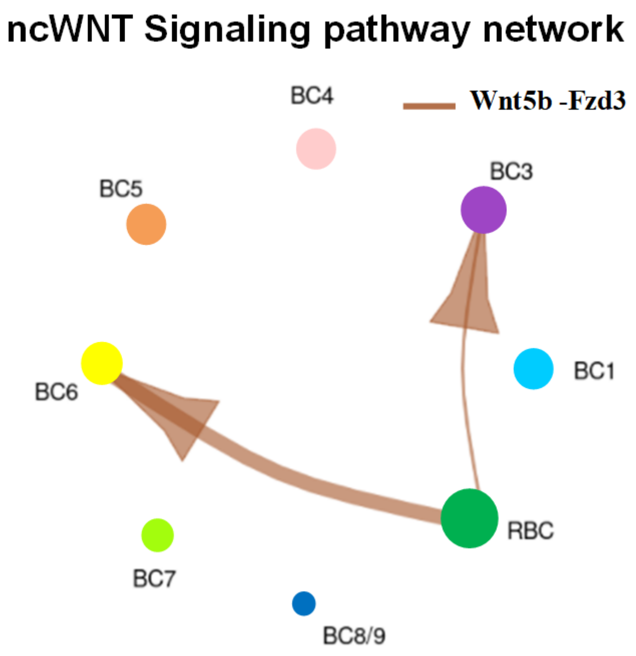

Supplementary Fig. 9. ncWNT signaling pathway among rare subpopulations of BC. A link between a filled circle (resource cluster with highly expressed ligand coding genes) and an unfilled circle (target cluster with highly expressed receptor coding genes) indicates the potential cell-cell communication of a signaling pathway. Circle colors represent different cell clusters, and the size represents the number of cells.

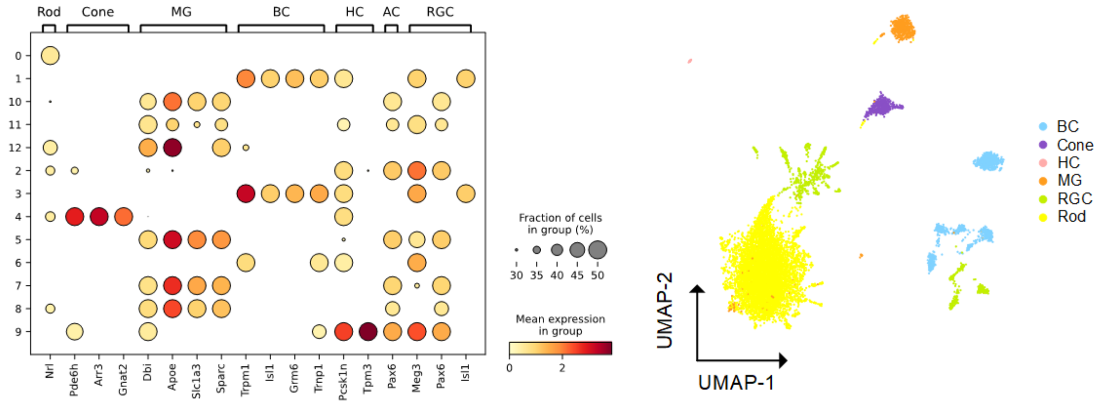

Supplementary Fig. 10. The results for GiniClust on mouse retina datasets. We used the same marker genes with MarsGT and annotated the cell populations.

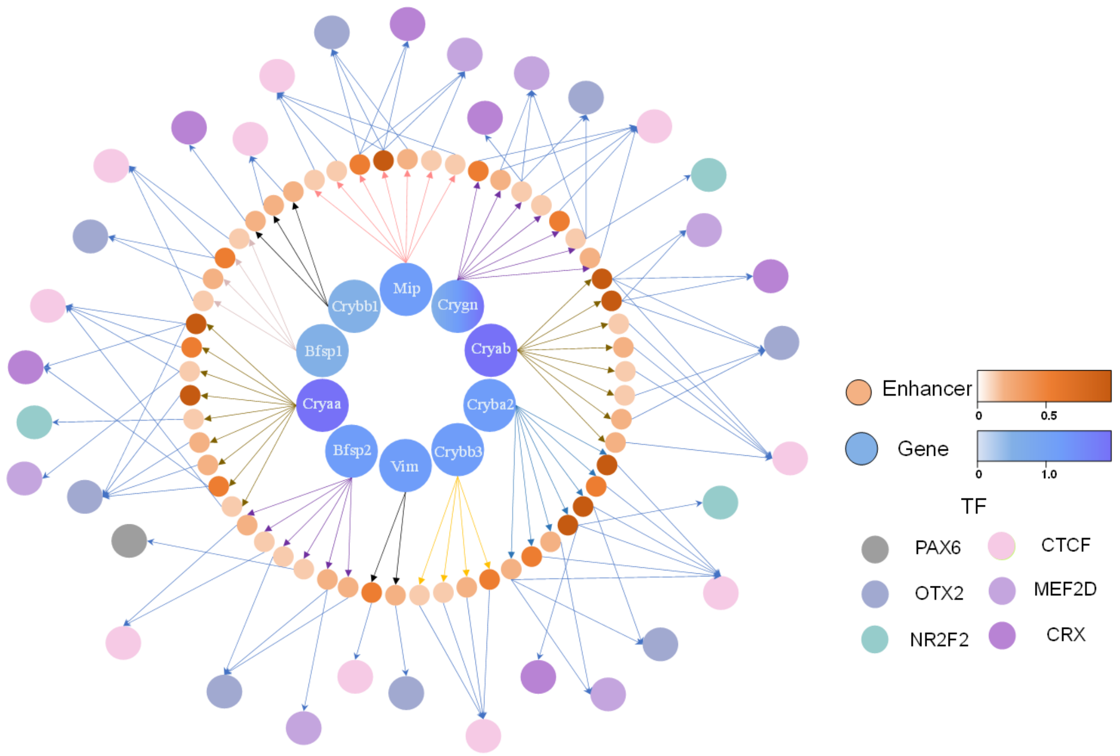

Supplementary Fig. 11. The eGRN of the structural constituent of eye lens pathway. The outer circles represent TFs. The inner circles represent genes in the structural constituent of eye lens pathway. The intermediate circles represent enhancers. The color of genes/enhancers represents the expression/accessibility.

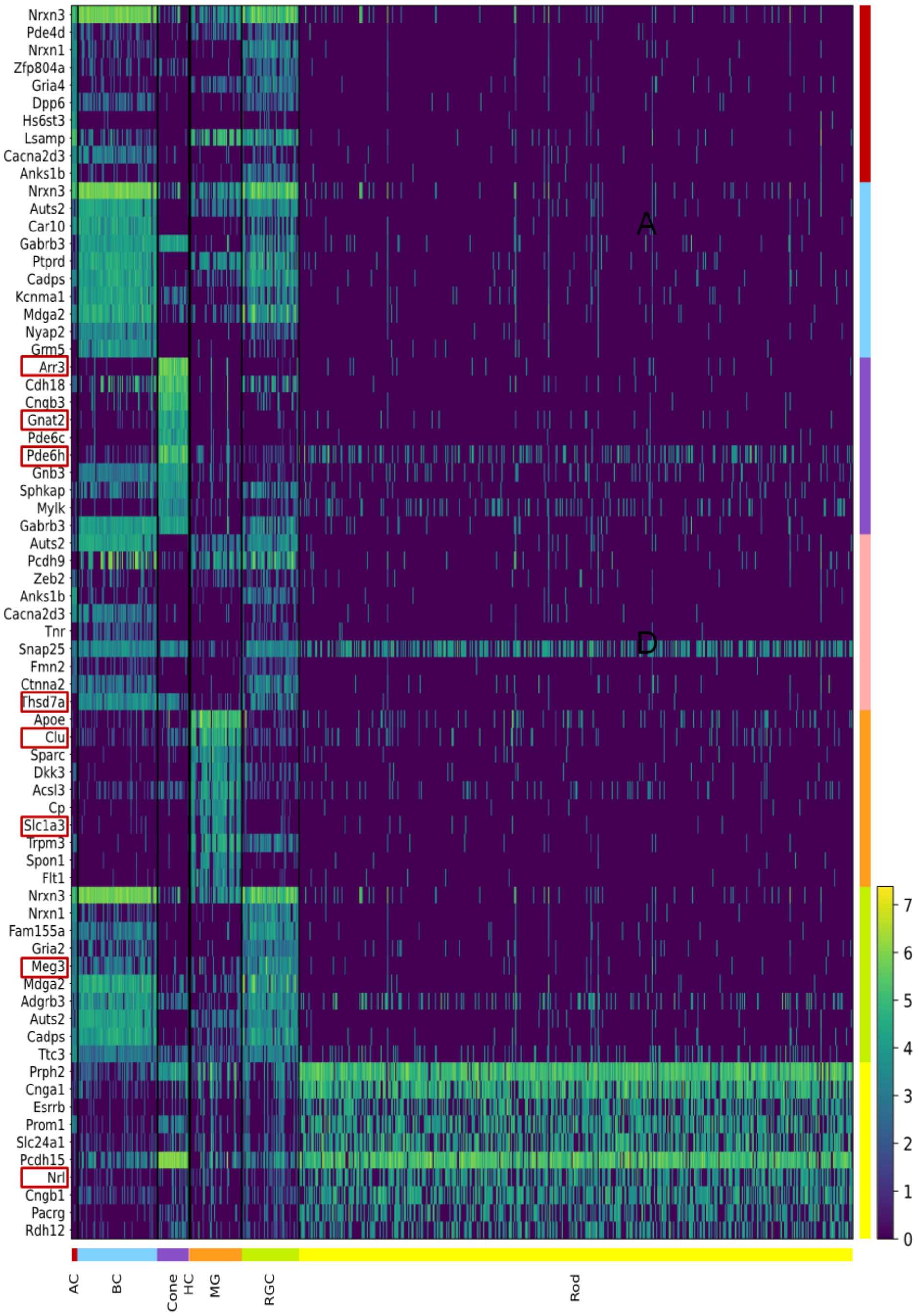

Supplementary Fig. 12. Heatmap of DEGs among all cell populations. The red box represents marker genes that have been reported by previous studies.

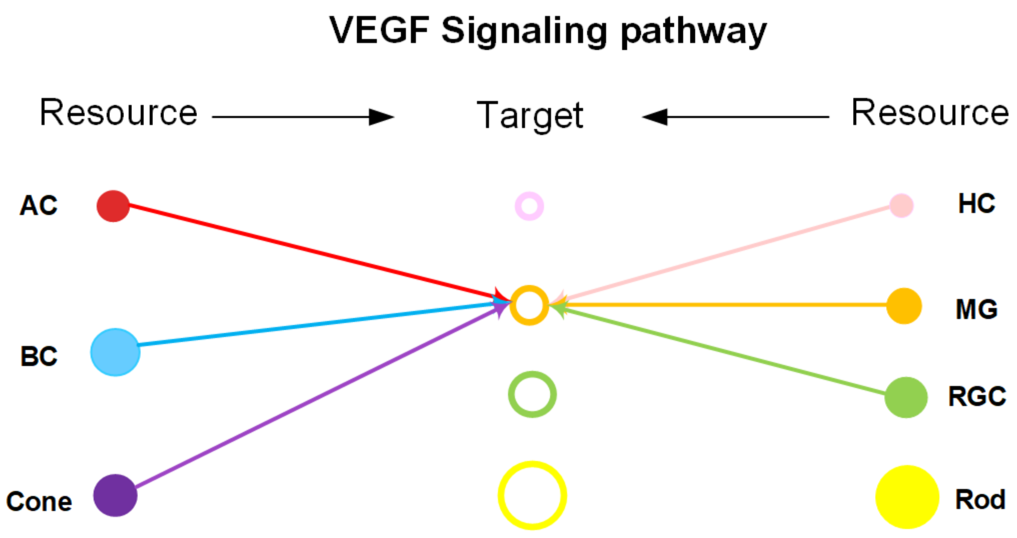

Supplementary Fig. 13. VEGF signaling pathway among major cell populations. A link between a filled circle (resource cluster with highly expressed ligand coding genes) and an unfilled circle (target cluster with highly expressed receptor coding genes) indicates the potential cell-cell communication of a signaling pathway. Circle colors represent different cell clusters, and the size represents the number of cells.

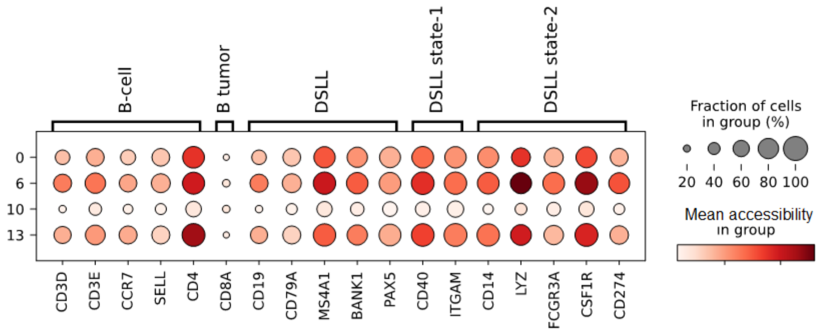

Supplementary Fig. 14. The dotplot of the accessibility of marker genes corresponding to enhancers. The size of the dot means the fraction of cells, and the color represents the accessibility in the group.

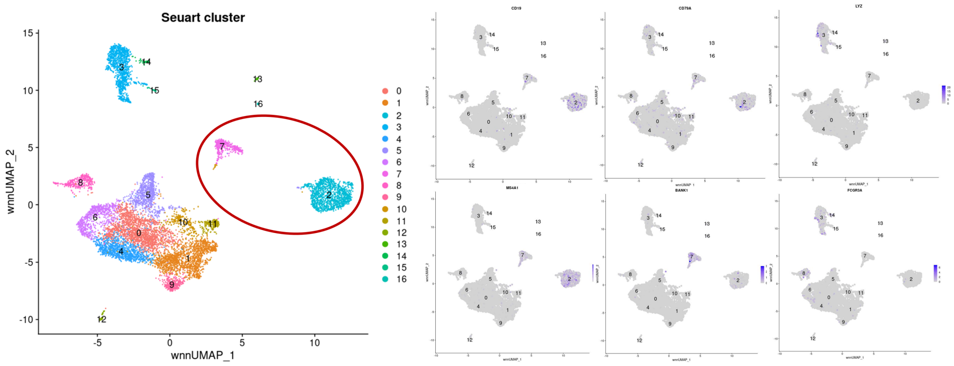

Supplementary Fig. 15. The cell cluster results by Seurat, the circle means B cells which are annotated by the curated marker genes.

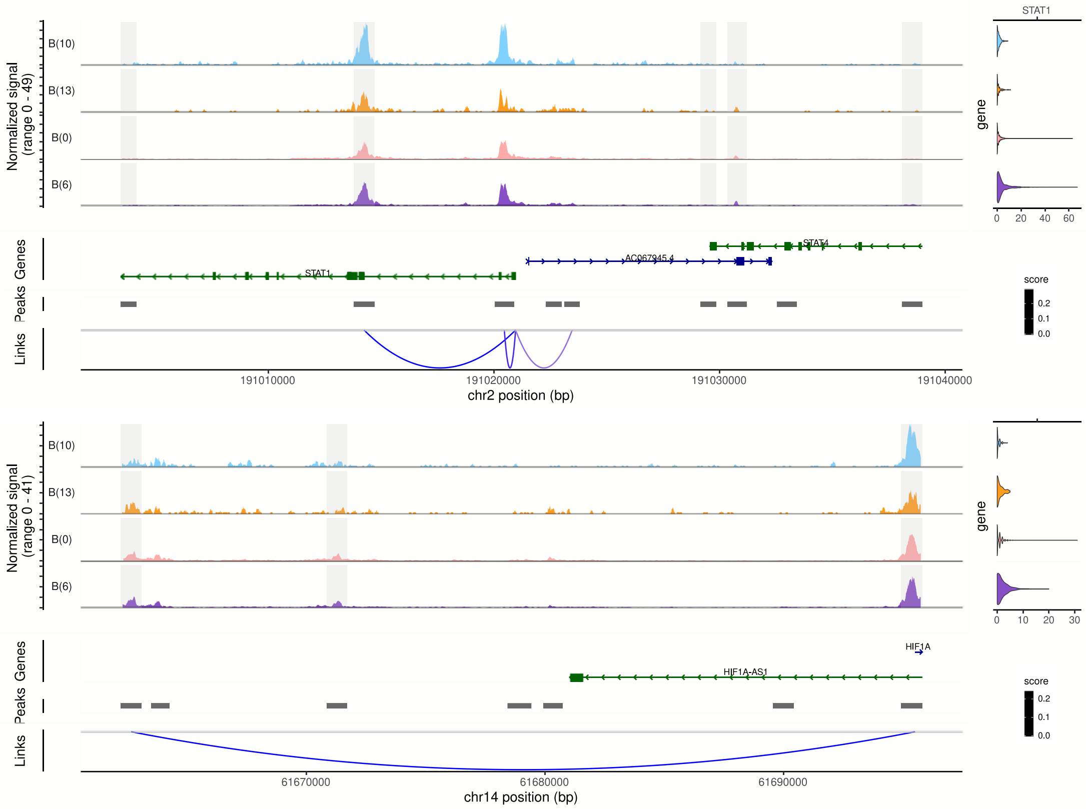

Supplementary Fig. 16. The Coverage plot for gene STAT1 and HIF1A.

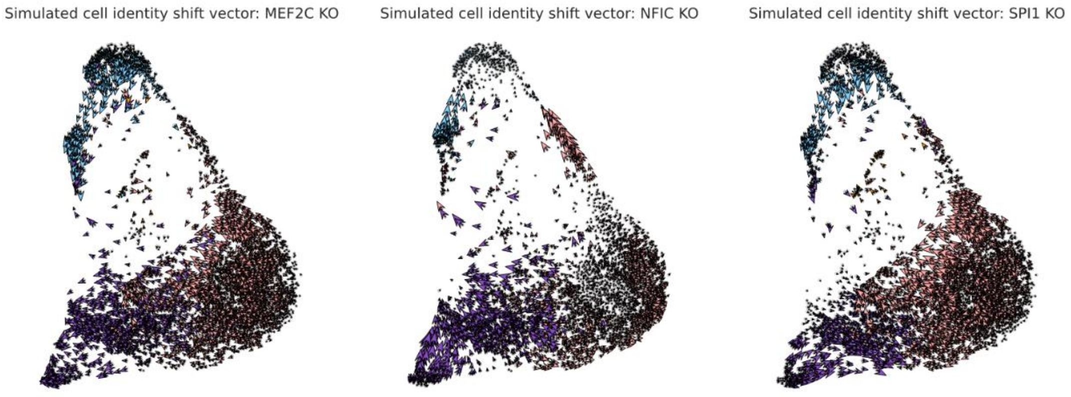

Supplementary Fig. 17. The observed and extrapolated future states (arrows) after the knockout of MEF2C, NFIC, and SPI1 on the four subtypes of B cells. The color represents the cell clusters.

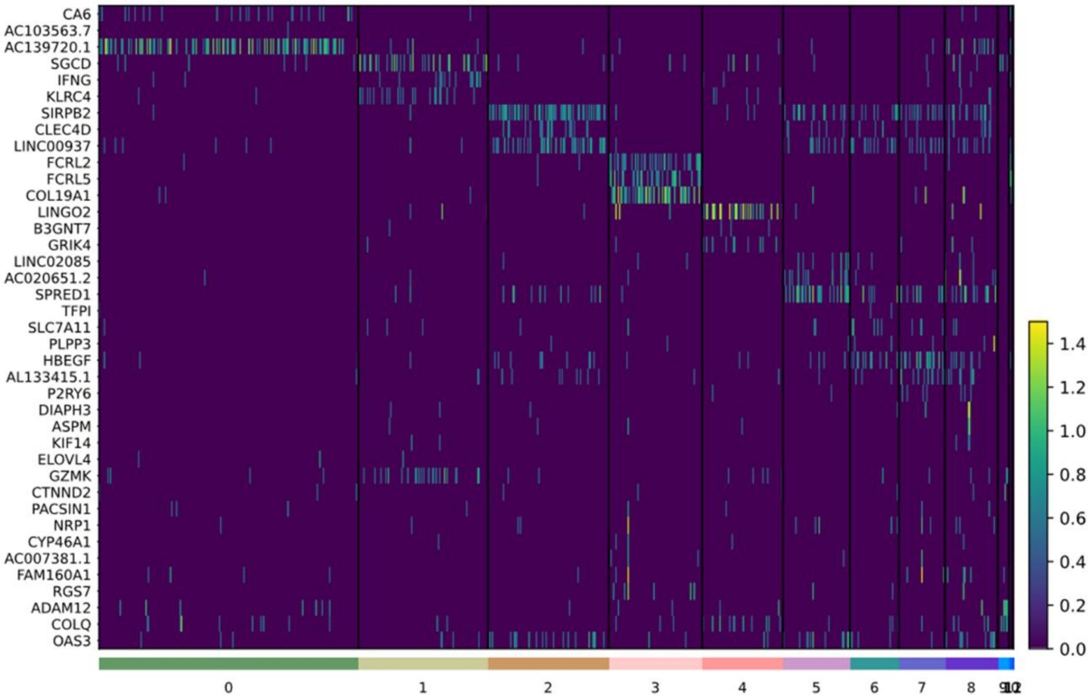

Supplementary Fig. 18. The heatmap of DEGs expression in each cell type.

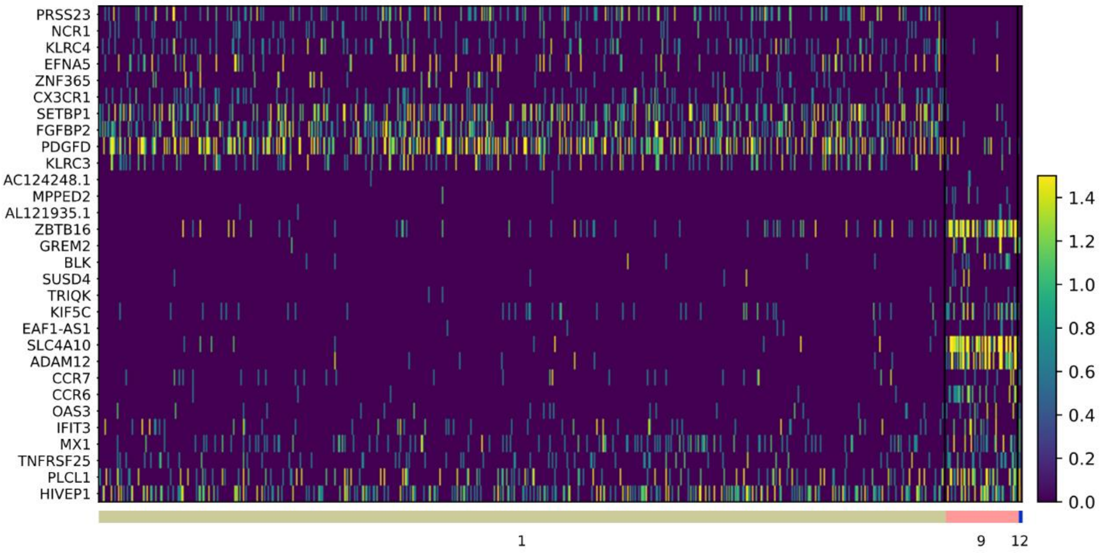

Supplementary Fig. 19. The heatmap of DEGs expression in three CD8+ cell types.

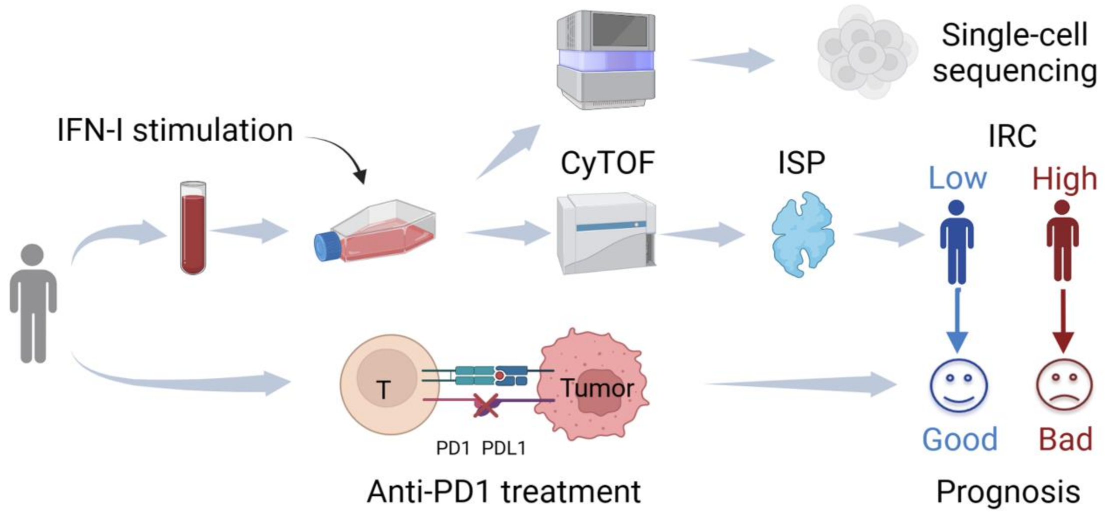

Supplementary Fig. 20. The introduction of the data information. Figure created with BioRender.com.

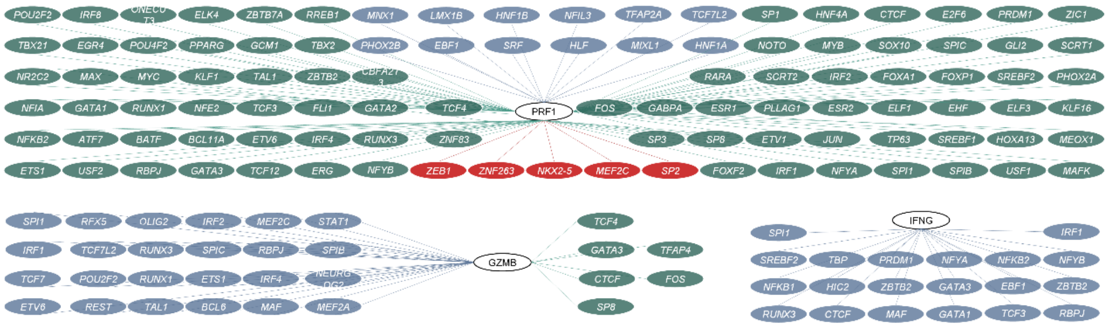

Supplementary Fig. 21. The regulatory relations of gene PRF1, GZMB, and IFNG. The red ellipse means the regulatory relation only exists in the high IRC, the blue ellipse means the regulatory relation only exists in the low IRC, and the green ellipse means the common regulatory relation between the high IRC and low IRC.

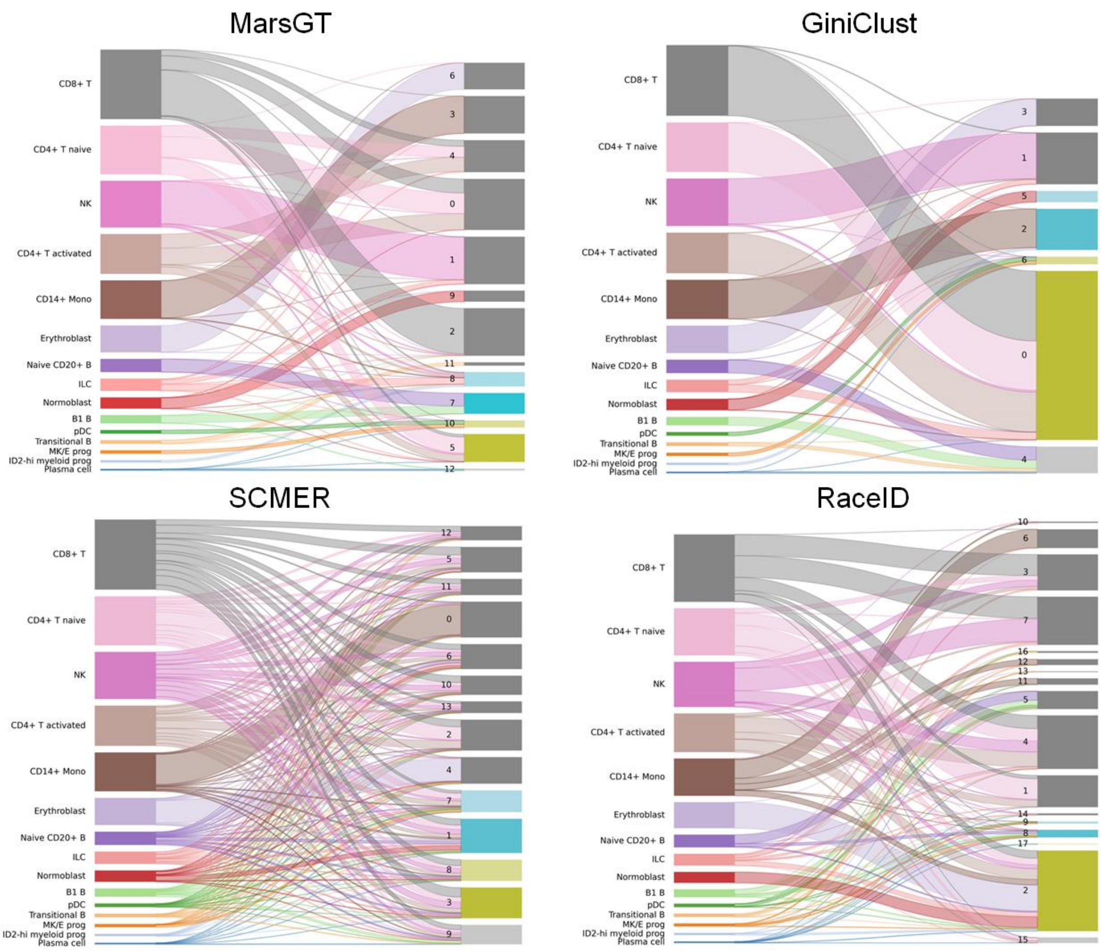

Supplementary Fig. 22. The Sankey plot of MarsGT, GiniClust, SCMER and RaceID on a simulation dataset. The left legend of each Sankey plot is the true label of the simulation dataset, and the right legend of each Sankey plot is the predicted label of the corresponding tool.

Supplementary Table S1: Cell Line and PBMC simulated Datasets

Dataset Type	Common Cell Types	Rare Cell Types
Sim-CL 1	PDX1, PDX2 (290 cells)	HeLa.S3 (10 cells)
Sim-CL 2	PDX1, PDX2 (280 cells)	HeLa.S3 (10 cells), K562 (10 cells)
Sim-PBMC 1	CD8+T (490 cells)	Plasma (10 cells)
Sim-PBMC 2	CD4+T naïve (480 cells)	HSC (10 cells), Plasma (10 cells)
Sim-PBMC 3	CD8+T (490 cells)	Erythroblast (10 cells)
Sim-PBMC 4	CD8+T (480 cells)	Erythroblast (10 cells), HSC (10 cells)
Sim-PBMC 5	CD8+T (480 cells)	Erythroblast (10 cells), Naive CD20+B (10 cells)
Sim-PBMC 6	CD14+Mono (250 cells), CD8+ (250 cells)	-
