# Supplementary material for: MarsGT: Multi-omics analysis for rare population inference using single-cell graph transformer
Source: Nat Commun. 2024 Jan 6;15:338. doi: 10.1038/s41467-023-44570-8 (PMC10771517; doi:10.1038/s41467-023-44570-8)
Supplement: Supplementary file 3 — Description of Additional Supplementary Files [file 41467_2023_44570_MOESM3_ESM.pdf]

Description of Additional Supplementary Files:

Supplementary Data 1: Datasets used in this paper for benchmarking and case study.

Supplementary Data 2: The evaluation of simulation datasets for benchmarking.

Supplementary Data 3: The evaluation of simulation datasets for the false positive test.

Supplementary Data 4: The evaluation of real datasets in different percentages of rare cell populations for benchmarking.

Supplementary Data 5: Grid optimization MarsGT on real bench sets.

Supplementary Data 6: The evaluation of real datasets for benchmarking.

Supplementary Data 7: Twenty repeated tests for robustness.

Supplementary Data 8: The cell-cell communication among BC types (case 1).

Supplementary Data 9: The enhancer-gene network with "the structural constituent of eye lens" pathway (case 1).

Supplementary Data 10: The enhancer-gene network in cell type 2 and cell type 10 (case 1).

Supplementary Data 11: The difference enhancer-gene network in cluster 2 and cluster 10 (case 1).

Supplementary Data 12: Top 10 DEGs in each cell type (case 1).

Supplementary Data 13: The cell-cell communication among major cell types (case 1).

Supplementary Data 14: The gene signature in three pathways (case 3).

Supplementary Data 15: The pathway enrichment score in each B cell type (case 2).

Supplementary Data 16: The enhancer-gene network in each B cell type (case 2).

Supplementary Data 17: The DEGs in clusters 1, 9, and 12 (case 3).

Supplementary Data 18: The marker gene signature of NKT and MAIT (case 3).

Supplementary Data 19: The enhancer-gene network in clusters 1, 9, and 12 (case 3).

Supplementary Data 20: The marker gene signature of exhausted and effector (case 3).
