# Supplementary material for: MarsGT: Multi-omics analysis for rare population inference using single-cell graph transformer
Source: Nat Commun. 2024 Jan 6;15:338. doi: 10.1038/s41467-023-44570-8 (PMC10771517; doi:10.1038/s41467-023-44570-8)
Supplement: Supplementary file 5 — Reporting Summary [file 41467_2023_44570_MOESM5_ESM.pdf]

nature portfolio

Corresponding author(s): Bingqiang Liu, Qin Ma

Last updated by author(s): Nov 30, 2023

Reporting Summary

Nature Portfolio wishes to improve the reproducibility of the work that we publish. This form provides structure for consistency and transparency in reporting. For further information on Nature Portfolio policies, see our Editorial Policies and the Editorial Policy Checklist.

Statistics

For all statistical analyses, confirm that the following items are present in the figure legend, table legend, main text, or Methods section.

n/a	Confirmed	
☐	☒	The exact sample size (n) for each experimental group/condition, given as a discrete number and unit of measurement
☐	☒	A statement on whether measurements were taken from distinct samples or whether the same sample was measured repeatedly
☐	☒	The statistical test(s) used AND whether they are one- or two-sided<br>*Only common tests should be described solely by name; describe more complex techniques in the Methods section.*
☒	☐	A description of all covariates tested
☐	☒	A description of any assumptions or corrections, such as tests of normality and adjustment for multiple comparisons
☐	☒	A full description of the statistical parameters including central tendency (e.g. means) or other basic estimates (e.g. regression coefficient) AND variation (e.g. standard deviation) or associated estimates of uncertainty (e.g. confidence intervals)
☐	☒	For null hypothesis testing, the test statistic (e.g. F, t, r) with confidence intervals, effect sizes, degrees of freedom and P value noted<br>*Give P values as exact values whenever suitable.*
☒	☐	For Bayesian analysis, information on the choice of priors and Markov chain Monte Carlo settings
☒	☐	For hierarchical and complex designs, identification of the appropriate level for tests and full reporting of outcomes
☒	☐	Estimates of effect sizes (e.g. Cohen's d, Pearson's r), indicating how they were calculated

Our web collection on statistics for biologists contains articles on many of the points above.

Software and code

Policy information about availability of computer code

Data collection	No software was used for data collection
Data analysis	MarsGT is open source and publicly available at https://github.com/mtduan/marsgt. Other tools and packages used in this paper include: python: 3.8.0, pytorch: 1.12.0, torch_cluster: 1.6.0, torch_scatter-2.1.0, torch_sparse:0.6.16 Seurat: 4.0.0, FiRE: 1.0.1, GapClust: 0.1.0, CellSIUS: 1.0.0, RaceID: 0.2.3, GiniClust: 3.0, SCMER: 0.1.0a3, scPower:1.0.0, CellOracle: 0.15.0. Zenodo link is https://doi.org/10.5281/zenodo.8406470.

For manuscripts utilizing custom algorithms or software that are central to the research but not yet described in published literature, software must be made available to editors and reviewers. We strongly encourage code deposition in a community repository (e.g. GitHub). See the Nature Portfolio guidelines for submitting code & software for further information.

Data

Policy information about availability of data

All manuscripts must include a data availability statement. This statement should provide the following information, where applicable:

- Accession codes, unique identifiers, or web links for publicly available datasets
- A description of any restrictions on data availability
- For clinical datasets or third party data, please ensure that the statement adheres to our policy

All data used for benchmarking and case study are collected from the public domain and can be retrieved using links or accession numbers provided in Supplementary Data 1.

Field-specific reporting

Please select the one below that is the best fit for your research. If you are not sure, read the appropriate sections before making your selection.

- [x] Life sciences
- [ ] Behavioural & social sciences
- [ ] Ecological, evolutionary & environmental sciences

For a reference copy of the document with all sections, see nature.com/documents/nr-reporting-summary-flat.pdf

Life sciences study design

All studies must disclose on these points even when the disclosure is negative.

Sample size	We analyzed 8 publicly available matched single-cell RNA-seq and single-cell ATAC-seq datasets. The cell number in each datasets are provided in Supplementary Data 1. Construct 550 simulated datasets. In our case analysis, we utilized scPower, a tool that assists in determining the power needed to detect a sufficient number of cells from a specific cell type in each individual. Using this tool, we calculated the minimum number of cells per individual required to reach a predetermined power threshold. This calculation was based on the negative binomial distribution.
Data exclusions	All cells and genes/peaks of each single-cell data set were used, and no exclusion was done prior to analysis.
Replication	We did not perform biological or technical replications. All data are collected from public domain. All computational replications for the same dataset were successful using the .h5ad ,provided in the Github. And the reproducibility is described in methods, "To evaluate the stability and reproducibility of MarsGT, we executed the algorithm 20 times on the independent test dataset, utilizing the default parameters. We then computed the variance of several key metrics, including the F1 score, precision, recall score, Normalized Mutual Information (NMI), purity, and entropy."
Randomization	Randomization is not relevant to this study since each data was analyzed separately. The analyses reported in this paper do not involve between sample comparison.
Blinding	Blinding is not relevant since no data collection was involved in the present study. Data information was provided alone with the original publication.

Reporting for specific materials, systems and methods

We require information from authors about some types of materials, experimental systems and methods used in many studies. Here, indicate whether each material, system or method listed is relevant to your study. If you are not sure if a list item applies to your research, read the appropriate section before selecting a response.

Materials & experimental systems

n/a	Involved in the study	
x		Antibodies
x		Eukaryotic cell lines
x		Palaeontology and archaeology
x		Animals and other organisms
x		Human research participants
x		Clinical data
x		Dual use research of concern

Methods

n/a	Involved in the study	
x		ChIP-seq
x		Flow cytometry
x		MRI-based neuroimaging
